# Fatal Errors

**Published:** 1881-10

**Authors:** Ad. Lippe

**Affiliations:** Philadelphia


### FATAL ERRORS.

AD. LIPPE, M. D., PHILADELPHIA.

IT is a fatal error to declare homœopathy and eclecticism synonyms, or to declare eclectics to be homœopathists.

This fatal error was committed by the President of the late International Convention, held in London, when he said in his address, July 12th, 1881:

> "If they [the propagandists] are habitually resorting to measures of another kind, using the stimulants and sedatives, the purgatives, the caustics and counter-irritants of ordinary medicine, their success, whatever it may be, makes nothing in the direction of our present outlook. But that they have perfect liberty so to do, if they think fit, I do not deny; on the contrary, I claim it for them. It is the supreme duty of us all to do what we judge best for our patients, irrespective of any creed or system, and to do this our hands must be free. We protest against the tyranny which ostracizes us because we believe this 'test' ordinarily to be homœopathy; and we will not be entangled again by any other yoke of bondage. No one may impugn our right of unfettered therapeutic choice—neither of our opponents, nor our stricter colleagues, nor our patients. Our only overt peculiarity is that we ally ourselves to institutions known as 'homœopathic,' to societies, dispensaries, and such like, which exist because of the exclusion of the method of Hahnemann and its practitioners from professional fellowship. We do not, by so acting, pledge ourselves to any exclusiveness in practice. We manfully recognize a truth which has laid hold of us, but which at present is denied and cast out. We in no way determine how far its practical consequences shall reach. What ground, then, have our enemies for charging us with inconsistency, with dishonesty, with trading in a name, if we use as freely as we think necessary the resources of ordinary medicine? With what propriety can friends, whose practice is more exclusive, reproach us with disloyalty for so doing? And as for our patients, they are as free to choose their doctor as he to select his remedies. They may come to him because he believes in homœopathy, but it is not their right, and, indeed, not their wisdom, to dictate to him how far his belief shall influence his remedial measures. If his treatment is not so purely homœopathic as they could wish, they have but to choose a practitioner more to their mind."

Thanks, sincere thanks to the learned gentleman who uttered these sentences. Under stress of circumstances he has at last taken off the mask, and allowed the profession to behold his true inwardness. We now know where to find him; not only has he fully exposed his untenable position, *i. e.*, that of a man who demands the indisputable right to practice eclecticism, and at the same time call himself a homœopath, but he also involuntarily proves correct, without a doubt, the proposition held by the International Hahnemannian Association, that there are numbers of professed homœopathists who violate the tenets of our school, and also largely repudiate them. He has furthermore done us, as a school, great service in exposing that "Nigger," supposed to be hidden in that woodpile, *i. e.*, the motives for this singular position assumed by him and the other eclectics.

The eloquent orator claims it to be the right of members of the homœopathic school of medicine to exercise perfect liberty in resorting to measures of any kind, to use "the stimulants and sedatives, the purgatives, the caustics and counter-irritants of ordinary medicine," because "it is the supreme duty of us all to do what we judge best for our patients, irrespective of any creed or system." It is our unpleasant duty to differ entirely with this learned expounder of the supreme duty of a professed homœopathist; what he proposes is simply eclecticism, and nothing else. Every member of the medical profession has an unquestionable right to do what he, with the light and knowledge he possesses, thinks best for his patient, but the learned orator forgets that the healing art, as introduced by Hahnemann, and by him called Homœopathy, admits of, nor *needs*, no such practices as are the prerogative of eclectics only. Nobody impugns the right of unfettered therapeutic choice; to deny that right would be tyranny indeed; but it is worse than tyranny to demand that consistent homœopathists must and shall fully indorse the eclectic tenets promulgated so plainly by this orator. When a medical man professes to practice homœopathy he voluntarily, and with his eyes wide open, submits himself to a yoke of bondage; he professes to be governed by the strict tenets of the school he embraces; he has no liberty to go outside of these tenets (while a member of the school), and such has been the time-honored course pursued by *all* the early pioneers of our school. By strict adherence to these principles they were able to give homœopathy the status it now enjoys among a host of intelligent people; none of these veterans ever thought it necessary to resort to the injurious means used by the ordinary medical profession.

When at this convention a notorious eclectic exclaimed, "Give me the *young men* to instruct, and I will guarantee the future of homœopathy!" the venerable Dr. Dunn called upon the *younger* men to be faithful to the truth, and not to remove the old land-marks that had been to him a guide through a long and successful career. Give

Dr. Dunn the young men to instruct, and the future of homœopathy would be glorious.

The learned orator admits that the hybrids, whom he represents, are charged by enemies "with inconsistency, with dishonesty, with trading in a name, if we use as freely as we think necessary the resources of ordinary medicine;" and are also charged "by friends, whose practice is more exclusive, with disloyalty for so doing." He pleads that they have no ground for so charging, and are guilty of an impropriety in so doing. The charge has been made, the facts have all been admitted, but enemies and friends have no reason for making this charge with propriety! Modest, indeed! very modest! The whole plea of the orator is, in sum and substance, that neither allopaths nor homœopaths, not even intelligent laymen when in need of a physician, have any right to know what are the tenets of the homœopathic healing art; they have no right to accept Hahnemann's teachings, nor heed the overwhelming testimony of the many true and faithful men who have successfully applied practically these tenets, these rules and regulations, and found them always sufficient, true and reliable in every respect. Time and again the learned orator and his friends have been asked to read "Blackstone on Evidence." Is not the positive testimony of one expert stronger evidence than the testimony of a hundred men who are ignorant of the point in dispute? Are, then, we again ask, the tenets of the homœopathic school an unerring guide in the healing art? The experts, who for a life-time have, like Dr. Dunn, been guided by the old land-marks through a long and successful career, those good and true men, gone before us, as well as many now living, willingly and gladly answer this question in the affirmative; while men who, by their own confessions and utterances, never, I say *never!* appreciated these tenets, who *never* applied them properly, intelligently or diligently, answer in the negative. Time and again have these men, testifying in the negative, been asked to *illustrate;* never, I repeat *never*, has any one of them related or reported a case of sickness treated by him under strict homœopathic law, rules and regulations without success; or after such failure became apparent, had been *cured* by means of "ordinary medicine." Till they, or any one of them, have done so, their negative testimony is utterly worthless. There is still a better mode of proving the superiority of the hybrid practice over strict homœopathy, a mode so easily of application, and that is, let these hybrids and eclectics publish their mortality list, and let the consistent homœopathists do likewise; the final results of these opposite practices would show in plain figures whose success is overwhelmingly greater. Sir John Forbes did that very thing, and his figures showed that homœopathy, then not so beset by departures as now, cured better, diminished the mortality list, shortened the time of disease, compared with the results the ordinary practice produced. Till then the allopathists and homœopathists will repeat their charges, because they know them to be just and true, and the mere bluster of an orator, who must have taken lessons from the speakers at "Belleville," will be ignored by all intelligent physicians of both schools, and by the people.

The orator finally removes the woodpile, and there is the "Nigger" to be sure. A unique character of Shakespeare exclaimed, "*Put money in thy purse.*" The orator says, "And as for our patients, they are as free to choose their doctors as he to select his remedies." That is true if he, the doctor, has been chosen because he professes to practice homœopathy, it is for the doctor to select the *homœopathic remedy;* but the orator modifies this correct proposition when he continues, "*they* [*the patients*] *may come to him because he believes in homœopathy, but it is not their right, and, indeed, not their wisdom, to dictate to him how far his belief shall influence his remedial measures.*" Just so; the doctor professes to be a homœopath, and the patient in good faith consults him. Now the doctor proposes to administer, say a purgative, or, if the patient is in much pain, a hypodermic injection of morphia; is it not, in such cases, the height of wisdom for the deceived sick to *politely* dismiss the pretender? The orator thinks differently; the patient has no right to know what are the tenets of homœopathy; it is not wisdom on the patient's part to discern between a genuine and pretending homœopath; it is not wisdom to discern between *theory* and knowledge, and when the knowledge is wanting theory will influence the doctor's remedial agents, if even they belong to the ordinary school. The patient expects his doctor to possess the necessary knowledge to treat him homœopathically, by all events the pretender, although found out, puts money in his purse. And now winds up the generous freedom-loving pretender, and in an overflow of liberality he says: "*If his* [*the pretender's*] *treatment is not so purely homœopathic as they* [*the sick*] *could wish, they have but to choose a practitioner more to their mind.*" Sublimely insolent! The pretender *professed* to be a homœopathist, his treatment is not purely homœopathic, it is then eclectic, his treatment has been *unsuccessful, unsatisfactory, harm-bringing,* just because it was not purely homœopathic; there it is, this want of knowledge and the falling away from the old landmarks (often well-known to the sick) caused all the tribulations the doctor now experiences; he had no success, that is all there is of it. Again, according to the sublimely insolent assertion of this orator, the people have not the "right, and, indeed, it is not their wisdom" to understand the fundamental principles governing the healing art. Why? Because the eclectics "*trading in a name,*" would no longer *put money in their purse.* That is it! That is what we expected the "Nigger," who was hid in the woodpile, to say.

Hereafter the true, honest, painstaking healers will continue to show by their acts what homœopathy can do, and by and by the orator and his blustering companions will exclaim with Othello:

"Farewell! Othello's occupation 's gone."

